# Tobacco-derived and tobacco-free nicotine cause differential inflammatory cell influx and MMP-9 in mouse lung

**DOI:** 10.1186/s12931-023-02662-5

**Published:** 2024-01-23

**Authors:** Thomas Lamb, Gagandeep Kaur, Irfan Rahman

**Affiliations:** Department of Environmental Medicine, University of Rochester Medical Center, 601 Elmwood Avenue, Box 850, Rochester, NY 14642 USA

**Keywords:** E-cigarettes, Flavors, Synthetic nicotine (TFN), Natural nicotine (TDN), MMPs

## Abstract

**Background:**

Electronic nicotine delivery systems (ENDS) or electronic cigarettes (e-cigarettes) aerosolize an e-liquid composed of propylene glycol (PG) and vegetable glycerin (VG) as humectants, flavoring chemicals, and nicotine. Nicotine naturally occurs in two isomers R- and S-nicotine, with tobacco-derived nicotine (TDN) composed of S-nicotine, and tobacco-free/synthetic nicotine (TFN) composed of a racemic mixture of R- and S-nicotine. Currently, there is limited knowledge of the potential differences in the toxicity of TFN versus TDN. We hypothesized that exposure of TFN and TDN salts to C57BL/6J mice would result in a differential response in lung inflammation and protease/ antiprotease imbalance.

**Methods:**

Five-week-old male and female C57BL/6J mice were exposed to air, PG/VG, PG/VG with TFN salts (TFN), or PG/VG with TDN salts (TDN) by nose-only exposure. Lung inflammatory cell counts, cytokine/chemokine levels, and matrix metalloproteinase (MMP) protein abundance and activity levels were determined by flow cytometry, ELISA, immunoblotting, and gel zymography, respectively.

**Results:**

Exposure to the humectants (PG/VG) alone increased cytokine levels- IL-6, KC, and MCP-1 in the BALF and KC levels in lung homogenate of exposed mice. While no change was observed in the cytokine levels in lung homogenate of TDN aerosol exposed mice, exposure to TFN aerosols resulted in an increase in KC levels in the lungs of these mice compared to air controls. Interestingly, exposure to TDN aerosols increased MMP-9 protein abundance in the lungs of female mice, while exposure to TFN aerosol showed no change. The metabolism of nicotine or the clearance of cotinine for TFN exposure may differ from that for TDN.

**Conclusion:**

Exposure to humectants, PG/VG alone, induces an inflammatory response in C57BL/6J mice. TFN and TDN salts show distinct changes in inflammatory responses and lung proteases on acute exposures. These data suggest variable toxicological profiles of the two forms of nicotine in vivo. Future work is thus warranted to delineate the harmful effects of synthetic/natural nicotine with humectants to determine the potential toxicological risks for users.

**Supplementary Information:**

The online version contains supplementary material available at 10.1186/s12931-023-02662-5.

## Background

Ever since the February 2020 ban by the US Food and Drug Administration (FDA) on the flavored cartridge-based electronic cigarettes (e-cigs), the national sales of disposable e-cigarette devices have increased drastically [1]. Shortly afterward, flavored products claiming to contain ‘tobacco-free nicotine’ (TFN) entered the markets. It is perceived that these products are; ‘cleaner’, ‘purer’, ‘tastier’, and ‘having higher quality’, as compared to traditionally available ‘tobacco-derived nicotine’ (TDN) products. While these perceptions have benefitted the sale of flavored e-cig products, they have also created confusion as many users thought them to contain ‘no tobacco or tobacco free’. Before April 2022, there was a regulatory gap that meant the FDA could not regulate TFN since it is not made or derived from tobacco, allowing tobacco companies to keep products on the market without needing to go through the pre-market approval process [1, 2]. In April 2022, this regulatory gap was closed after the new legislation allowed the FDA to regulate TFN products [1]. Yet, there is little to no knowledge with regards to the toxic effects of the use of TFN-containing products.

Nicotine is a naturally occurring alkaloid extracted from tobacco leaves that exists in two isomers: (R)-(+)-nicotine and (S)-(−)-nicotine [3, 4]. Traditionally tobacco products have utilized nicotine extracted from tobacco leaves which contains predominantly S-nicotine [5, 6]. TFN, on the other hand, is a chemically synthesized nicotine containing a racemic (50:50) mixture of R- and S-forms [5].

Besides these two isomers, nicotine is present in two forms- a protonated and a free base form [7]. The protonated form of nicotine is formed from the addition of acid to freebase nicotine to form nicotine salts. The two most commonly used acids for this purpose being lactic and benzoic acid [8]. Nicotine salts in e-liquids are found to have a lower pH level than freebase nicotine even with higher levels of nicotine present [9]. This reduction in pH is believed to reduce the throat irritation and harshness due to high levels of nicotine [10]. In clinical studies, protonated nicotine has been found to result in a higher nicotine absorption than free-base nicotine, although, contrary evidence exists through in vivo and in vitro studies, thus warranting further investigation [7, 11].

Nicotine is known to have adverse effects on the respiratory system [12]. Previous work within our lab using freebase nicotine, has shown that acute exposure (3 days; 2 h/day) of C57BL/6J mice to PG with nicotine resulted in an increase in pro-inflammatory cytokines and matrix metalloproteinase 2 (MMP-2) levels in exposed mice as compared to the air controls [13]. Furthermore, sub-chronic (3 weeks; 3 h/d; 5 d/wk) exposures of C57BL/6J mice to PG/VG with freebase nicotine has also demonstrated sex-specific alterations in lipogenic and myogenic gene expression and levels of MMP-9 [14]. Our lab has also previously conducted research on e-cigarettes with nicotine salts, and has found pods containing nicotine salts result in an increase in pro-inflammatory cytokines, oxidative stress, epithelial barrier dysfunction and DNA damage in lung epithelial cells [15]. These results provide evidence for the adverse toxicological effects of both freebase and protonated form of nicotine. However, there is limited knowledge about the pharmacological effects of R-nicotine. Current data has indicated that R-nicotine is a less potent agonist of nicotinic acetylcholine receptors and can bind and inhibit acetylcholinesterase. Further, R-nicotine and R-cotinine have a faster clearance than S-nicotine or S-cotinine [5, 16, 17].

We thus hypothesized that exposure of C57BL/6J mice to TFN salts will result in a differential response in lung inflammation and protease/antiprotease imbalance compared to TDN salts exposed mice. To test this hypothesis, we exposed mice to air, PG/VG, PG/VG with TFN salts, or PG/VG with TDN salts, and measured the cytokine levels in BALF and lung homogenate along with MMP protein abundance in the lungs of exposed mice.

## Materials and methods

### Ethics statement

Experiments were conducted using the standards established by the United States Animal Welfare Act. Animal experimental protocols conducted at the University of Rochester were approved by the University Committee on Animal Resources. All laboratory studies were approved by the Institutional Biosafety Committee of the University of Rochester Medical Center.

### Mouse exposures

An equal number of male (12) and female (12) C57BL/6J mice at 5 weeks old were ordered from Jackson Laboratory. Mice were housed for 1 week at the University of Rochester Vivarium prior to being moved to the inhalation suite to begin nose-only tower training. In order to acclimatize the mice to the mesh restraints of the nose-only tower, 1 week prior to beginning the e-cig exposure, mice were placed in the mesh restraints and held in the tower. Mice were trained for 5 days following the methodology described in Lamb et al. [18]. The first day mice were held in the restraints for 15 min, the second day mice were held in the restraints for 30 min, the third day mice were held in the restraints for 45 min, and the final 2 days mice were held in the restraints for 1 h.

### E-cigarette device and e-liquid

A Joyetech eVic-VTC mini and cubis pro atomizer (SCIREQ, Montreal, Canada), with a BF SS316 1.0 Ω coil (Joyetech, Shenzhen, China) and the Scireq nose-only tower (SCIREQ, Montreal, Canada) were utilized for all e-cigarette exposures. PG and VG from “EC Blend” were purchased through local vendors/online vendors. A 1:1 mixture of PG/VG was used for PG/VG exposures and a 1:1 mixture of PG/VG mixed with a 1:1 mixture of lactic acid with R/S-nicotine ((±)-Nicotine, Sigma-Aldrich, Cat# N0267) or S-nicotine ((−)-Nicotine, Sigma-Aldrich, Cat#N3876) at a concentration of 50 mg/mL was used for PG/VG with TFN salts and PG/VG with TDN salts exposure, respectively. Nicotine e-liquids utilized for exposures were analyzed by proton nuclear magnetic resonance (^1^H NMR) to confirm nicotine concentration and R/S ratio. In brief ^1^H NMR following the same basic methodology as described in Lamb, et al. was used to determine nicotine concentrations for PG/VG with TFN salts at 52.09 mg/mL and for PG/VG with TDN salts at 48.35 mg/mL [18]. The ratio of R-nicotine to S-nicotine was determined by ^1^H NMR following a similar methodology as described in Duell et al. and determined the R/S ratio of nicotine in PG/VG with TFN salts to be 56/44 and in PG/VG with TDN salts to be 0/100 [19].

### E-cigarette exposure

Nose-only e-cigarette exposure was conducted utilizing the Scireq InExpose system with the Scireq flexiware software controlling the Joyetech eVic-VTC mini device. Mice were exposed to a puffing profile of two puffs per minute with a puff volume of 51 mL, puff duration of three seconds, and an inter puff interval of twenty seven seconds with a 2 L/min bias flow between puffs [20]. Mice were split into four groups-(i) air, (ii) PG/VG, (iii) PG/VG with 50 mg/mL TDN salts (labelled as TDN), and (iv) PG/VG with 50 mg/mL TFN salts (labelled as TFN), of equal number of male (*n* = 3) and female (*n* = 3) mice. Mice were exposed to the described puffing profile for 1 h per day (120 puffs) for a total of five days, with air mice being exposed to room air [21]. Temperature, humidity, and CO levels were measured at the starting, mid-, and end-point of the exposure utilizing Q-Trak Indoor Air Quality Monitor (TSI, SKU#7575). Total particulate matter (TPM) measurements were taken at the exhaust tubing at the half-way point of the exposure and at the inlet tubing connected to the top of the nose-only tower immediately after the end point of the exposure. TPM was measured by weighing a glass fiber filter pad (Pall Corporation, P/N#61630) before and after collecting aerosol over the course of five minutes. Cotinine levels in the blood serum of exposed mice were measured using an ELISA based assay (Calbiotech, Cat#C0096D) following manufacturer’s protocol.

### Mouse sacrifice

Mice were sacrificed 2 h after the final e-cigarette exposure and were anesthetized with a mixture of ketamine and xylazine. Blood was drawn from the inferior vena cava, and allowed to sit for roughly thirty minutes before being centrifuged at 2000 rpm for fifteen minutes. After centrifugation, the serum was collected and stored at − 80 °C. Manual lung perfusion was performed by taking 3 mL of 1 × PBS in a 3 mL syringe and slowly injecting the 1 × PBS into the heart of the mouse until the lung lobes turned white. Mice were lavaged via catherization three separate times with 0.6 mL of 0.05% FBS in 0.9% NaCl. The combined lavage fluids were centrifuged at 3000 rpm for 10 min at 4 °C. The supernatant was recovered and stored at − 80 °C until further experimentation, while the cell pellet was re-suspended in 1 mL of 1 × PBS for determination of immune cell population. Mouse lung lobes were harvested from exposed mice and washed with 1 × PBS, one lobe was left in 1 mL 1 × PBS while the rest were blotted dry using a filter pad, and then flash frozen by dry ice before being stored at − 80 °C.

### Lung digest, cell count, and flow cytometry

Lung lobes to be used for lung digestion were minced finely, and placed into a 50 mL conical tube with a liberase enzymatic cocktail (0.5 mL of 5 mg/mL liberase with 2 mL DMEM and 3 µL of 100 mg/mL DNAse I). Tissue samples were dissociated using gentleMACS Dissociator (Miltenyi, Biotec, Gaithersburg, MD, USA), placed on a rocker and incubated at 37 °C for thirty minutes. After incubation, cell suspensions were strained through a 70 micron cell strainer into a new 50 mL conical tube and then remaining cells were collected by adding 5 mL DMEM (10% FBS) through the cell strainer. Cells were centrifuged at 300 g for five minutes at 4 °C, afterwards, the supernatant was removed and 1 mL of RBC lysis buffer was added to the cell pellet and incubated on ice for one minute. After incubation 5 mL of DMEM (10% FBS) was added to the cell suspension and then centrifuged at 300 g for five minutes at 4 °C. Supernatant was removed and the cell pellet was re-suspended in 2 mL DMEM (10% FBS). Total cell counts for bronchoalveolar lavage fluid (BALF) and lung digest were measured by staining cells with Acridine Orange/Propidium Iodide (AO/PI) and counted using the Nexcelom Cellometer Auto 2000 cell viability counter. Differential cell counts were determined by flow cytometry using the BD LSRFortessa cell analyzer. Cells from both lung digest and BALF were stained with CD16/32 (Cat#70-0161-u500, Tonbo Biosciences, 1:10 dilution) to block nonspecific binding and then stained with a master mix of Siglec F (Cat#740280, BD Biosciences, 1:200 dilution), CD11b (Cat #101243, Biolegend, 1:200 dilution), Ly6G (Cat# 562700, BD Biosciences, 1:200 dilution), CD45 (Cat#103126, Biolegend, 1:200 dilution), and CD11c (Cat #117318, Biolegend, 1:200 dilution), with 7AAD (Cat#00-6993-50, eBiosciences, 1:10 dilution) being added just prior to analysis by flow cytometry with cells used for compensation.

### Protein extraction

Approximately 20–30 mg of flash frozen lung tissue was added to 350 µL of RIPA buffer containing protease inhibitor (Cat#87785, Thermo Fisher Scientific) and EDTA (Cat#R1021, Thermo Fisher Scientific) and mechanically homogenized while on ice. After homogenization, samples remained on ice for forty-five minutes and then spun at 14,000 rpm for thirty minutes at 4 °C. The supernatant was collected and 50 µL aliquots were stored at − 80 °C. Total protein concentration for each sample was determined using the Pierce BCA Protein Assay kit (Cat#23225, Thermo Fisher Scientific) with BSA being utilized as the protein standard.

### Pro-inflammatory cytokines/chemokines levels

Pro-inflammatory cytokine/chemokine keratinocyte chemoattractant (KC) (R&D DuoSet DY453), interleukin-6 (IL-6) (R&D Duoset DY406), and monocyte chemoattractant protein-1 (MCP-1) (R&D DuoSet DY479) levels were measured using ELISA following manufacturer’s protocol in BALF and lung homogenate. A dilution of 1:10 was utilized for lung homogenate samples and no dilution was utilized for BALF samples. Lung homogenate was normalized to total protein amount of each sample.

### Immunoblot assay

An equal concentration of protein (10 µg) from each lung homogenate samples were loaded per well of a 26 well 4–15% Criterion Precast Gel (Cat#5671085, BioRad) with 10 µL of Precision Plus Protein™ Kaleidoscope™ Prestained Protein Standards (Cat# #1610375, BioRad) added to the first well. Protein were separated based on size using gel electrophoresis before being transferred to a nitrocellulose membrane. Membranes were blocked for 1 h with 5% BSA in 1 × TBST or with 5% non-fat milk in 1x TBST at room temperature. Membranes were probed with primary antibodies, using the following antibodies diluted in 1% BSA in 1 × TBST: tissue inhibitor of metalloproteinases-1 (TIMP-1) (Cat#ab179580, Abcam, 1:1000), and the following antibodies diluted in 5% non-fat milk in 1 × TBST: MMP-2 (Cat# ab92536, Abcam, 1:1000), MMP-9 (Cat# ab38898, Abcam, 1:1000), and MMP-12 (Cat# NBP2-67344, Novus Biological, 1:1000) and were left rocking overnight at 4 °C. After membranes were incubated overnight, membranes were washed with 1x TBST and then incubated with a goat anti-rabbit secondary antibody (Cat#1706515, BioRad, 1:10,000) in 5% BSA in 1 × TBST or 5% non-fat milk in 1 × TBST. After incubation with secondary antibody, membranes were washed with 1 × TBST and then signals were measured using West Femto Maximum Sensitivity Substrate (Cat#34096, Thermo Fisher) following the manufacturer’s protocol. Images of membranes were collected utilizing the Bio-Rad ChemiDoc MP Imaging system (Bio-Rad Laboratories). After imaging, membranes were stripped utilizing restore western stripping buffer (Cat#21063, Thermo Fisher) and re-probed with antibodies for the other proteins and finally for GAPDH (Cat# 2118 S, Cell Signaling, 1:1000). Band intensity was determined using densitometry analysis using image lab software and normalized to the levels of GAPDH. Fold change in protein abundance were relative to the protein abundance of air-exposed mice.

### Gelatin gel zymography

Total MMP activity levels were measured by following the gelatin zymography protocol from Abcam with slight modifications. The night prior to the start of the assay, the gelatin gel was prepared with a 15-well comb utilizing the Mini-PROTEAN Tetra Cell Casting Module (Cat#1658022 BioRad). After the gels had solidified, the gels were left at 4 °C in 1 × running buffer. An equal concentration of protein (50 µg) from each lung homogenate samples were loaded per well of the gelatin gel, with 10 µL of Precision Kaleidoscope Prestained Protein Standards (Cat# #1610375, BioRad) added to the first well. Proteins were separated based on size through the stacking gel and separating gel. Once the protein are separated, the gels were washed with washing buffer two times for thirty minutes, rinsed twice with incubation buffer for 10 min per rinse while rocking, and then fresh incubation buffer was added to cover the gels which were then incubated for 24 h at 37 °C. After incubations, the gels were stained with staining solution for 1 h with rocking at room temperature. After staining, the gels were rinsed with ddH_2_O and then destaining solution was added and incubated until bands could be visualized. Images of membranes were collected utilizing the Bio-Rad ChemiDoc MP Imaging system (Bio-Rad Laboratories). After imaging, band intensity was determined using densitometry by image lab software, and fold change was determined relative to the activity levels of air-exposed mice.

### Statistical analysis

Analysis was performed using GraphPad Prisma utilizing One-Way ANOVA with Tukey’s multiple comparisons test with data shown as mean ± SEM.

## Results

### PG/VG with synthetic nicotine salts exposure alters inflammatory cells infiltration in exposed mice

In order to determine the effects of TFN and TDN salts on inflammatory cell influx in vivo, C57BL/6J mice were exposed to nose-only exposure to aerosols from PG/VG, TFN and TDN salts as described earlier. Average TPM measurements at the inlet for all the exposures was comparable with the values being: 2755.33 mg/m^3^ for PG/VG, 3300.67 mg/m^3^ for TFN, and 3367.00 mg/m^3^ for TDN exposures. We further performed Cotinine assay to ensure exposure to tobacco in our samples and found the levels of cotinine in the blood serum to be significantly varied between TFN (53.69 ± 9.63 ng/mL) and TDN (104.41 ± 28.09 ng/mL) exposed mice (Additional file 1: Fig. S1A).

**Fig. 1:**
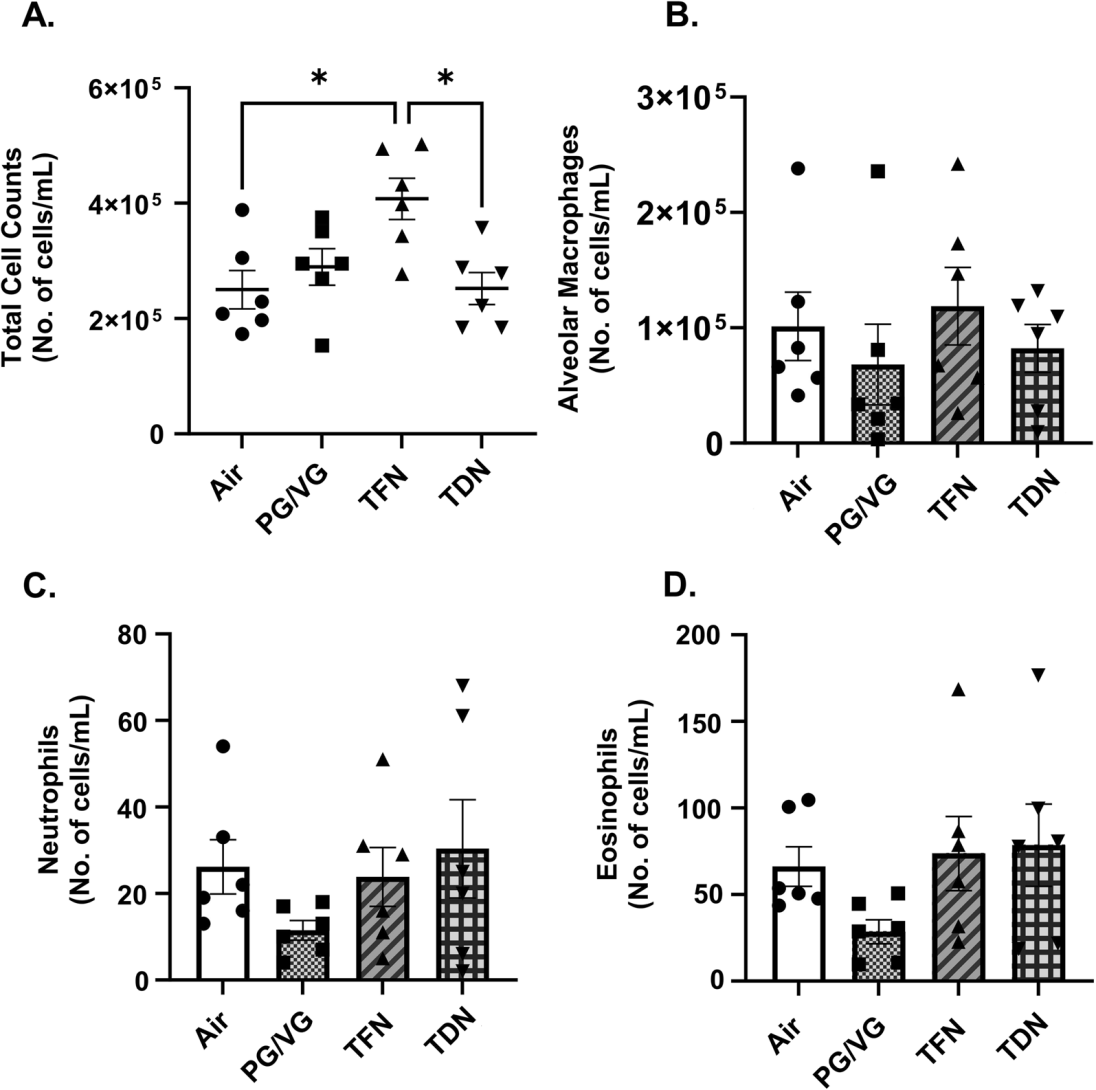
Differential effects of synthetic and tobacco-derived nicotine salts on infiltrating inflammatory cells in BALF. Mice were exposed to air, PG/VG, PG/VG with TFN salts (TFN), and PG/VG with TDN salts (TDN) for 5 days for 1 h per day. Mice were sacrificed 2 h after final exposure. **A** Total cell counts were measured in BALF by staining cells with AO/PI and counted with a cellometer. Flow cytometry was performed to determine the number of **B** Alveolar Macrophages (CD45^+^Siglec F^+^CD11b^−^), **C** Neutrophils (CD45^+^Siglec F^−^CD11b^+^Ly6G^+^), and **D** Eosinophils (CD45^+^CD11b^+^Ly6G^−^CD11c^−^Siglec F^+^) in BALF of control and experimental groups. Data represented as mean ± SEM and analyzed using one-way ANOVA with Tukey’s multiple comparison with * *p* < 0.05, *N* = 6/group

Lung inflammation following acute exposures to TFN and TDN salts was determined using flow cytometry. Interestingly, we observed a significant increase in the total cell counts in BALF from TFN exposed mice as compared to the mouse exposed to TDN salts pointing towards elicitation of varied immune responses in C57BL/6J mice on exposure to TFN salts versus TDN (Fig. 1A). Mice exposed to PG/VG, TFN salts, and TDN salts did not alter the differential cell counts of alveolar macrophage, eosinophils or neutrophils in the BALF compared to air exposed mice (Fig. 1B–D). We did not observe any change in the differential cell counts of alveolar macrophages, neutrophils and eosinophils in the lung homogenates from TFN and TDN exposed mouse lungs as compared to air controls as well. It is pertinent to mention, though, that the neutrophilic responses in the lung tissues of TFN exposed mice (73,487 ± 55,121 cells/mL) was higher than those exposed to TDN (28,523 ± 5202 cells/mL) (Additional file 1: Fig. S1B–D).

### PG/VG exposure alters inflammatory cytokines in BALF

**Fig. 2:**
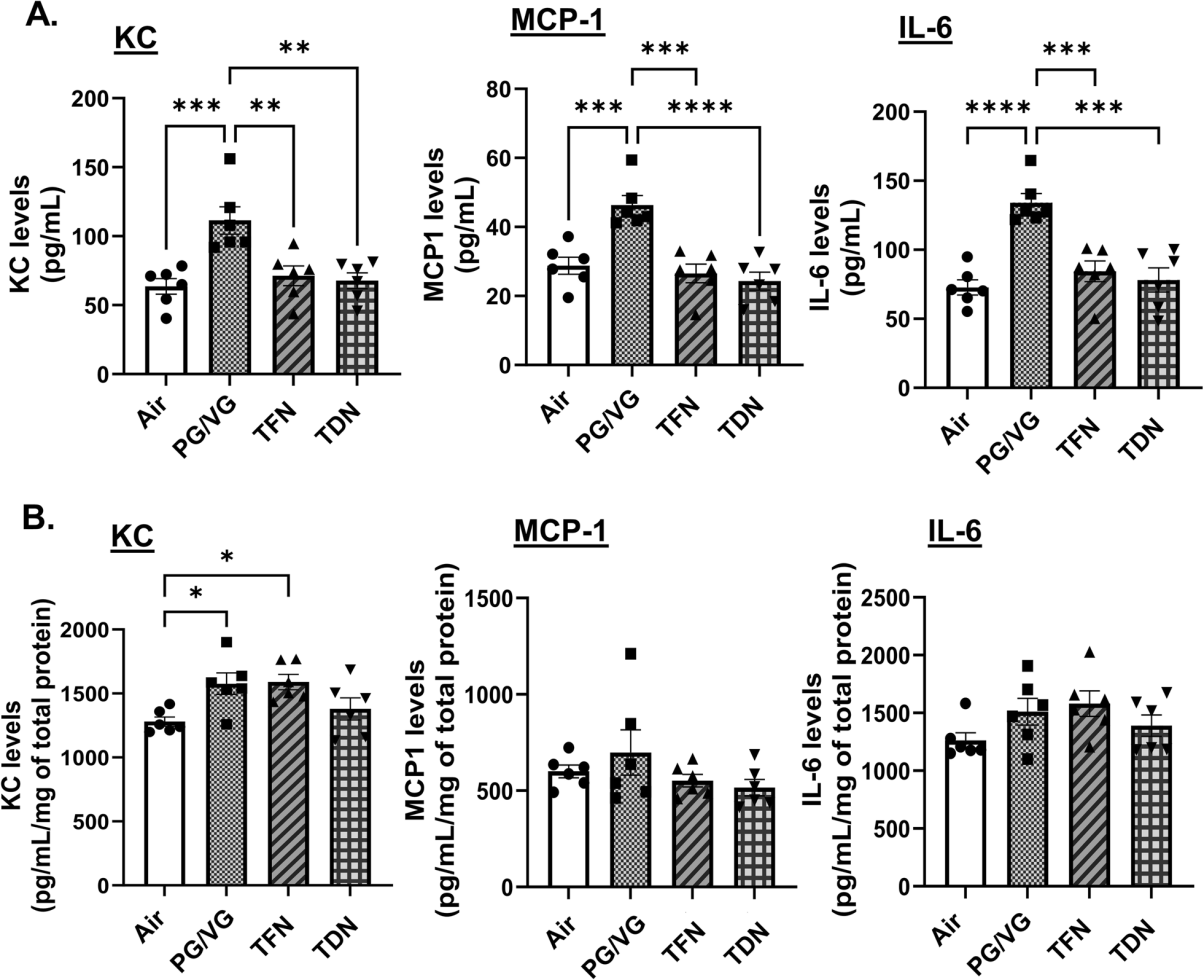
Differential effects of synthetic and tobacco-derived nicotine salts on pro-inflammatory cytokine production. Mice were exposed to air, PG/VG, PG/VG with TFN salts (TFN), and PG/VG with TDN salts (TDN) for five days for 1 h per day. Mice were sacrificed 2 h after final exposure. Pro-inflammatory cytokines, IL-6, KC, and MCP-1 were measured in (**A**) BALF and (**B**) lung homogenate. Data represented as mean ± SEM and analyzed using one-way ANOVA with Tukey’s multiple comparison with * *p* < 0.05, ** *p* < 0.01, *** *p* < 0.001, and **** *p* < 0.0001, *N* = 6/group

In order to determine the potential of TFN and TDN salt exposure to induce an inflammatory response, pro-inflammatory cytokine level was measured in BALF and lung homogenate of control and exposed mice. Mice exposed to PG/VG resulted in a significant increase in IL-6, KC, and MCP-1 levels in BALF compared to air, TFN, and TDN salts exposed mice (Fig. 2A). Contrarily, mice exposed to PG/VG, TFN, or TDN salts did not significantly alter IL-6 and MCP-1 levels in lung homogenate compared to air exposed mice (Fig. 2B). Mice exposed to PG/VG and TFN salts significantly increased lung KC levels as compared to air exposed mice (Fig. 2B).

### PG/VG with tobacco-derived nicotine salts alters MMP-9 protein abundance

**Fig. 3:**
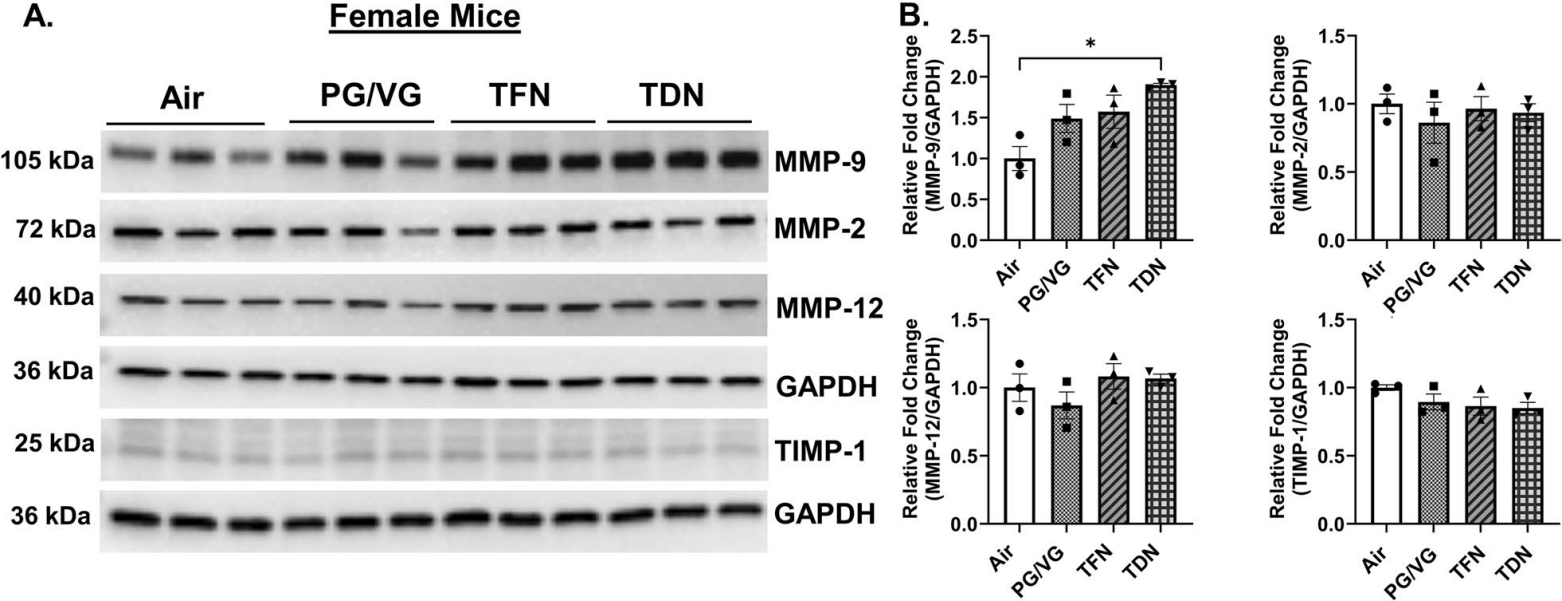
Differential effects of synthetic and tobacco-derived nicotine salts on MMPs protein abundance in female mice. Mice were exposed to air, PG/VG, PG/VG with TFN salts (TFN), and PG/VG with TDN salts (TDN) for five days for 1 h per day. Mice were sacrificed 2 h after final exposure. The protein abundance of MMP-2, MMP-9, MMP-12, and TIMP-1 was measured in lung homogenate with GAPDH used as a loading control by western blot. **A** Representative images for MMP-2, MMP-9, MMP-12, TIMP-1, and GAPDH for exposed female mice. **B** Band intensity was measured by densitometry with relative fold change being measured compared to air exposed female mice. Data represented as mean ± SEM and analyzed using one-way ANOVA with Tukey’s multiple comparison with * *p* < 0.05, *N* = 3/group. Images of full blots are shown in Additional file 1: Figs. S2A–S7A

**Fig. 4:**
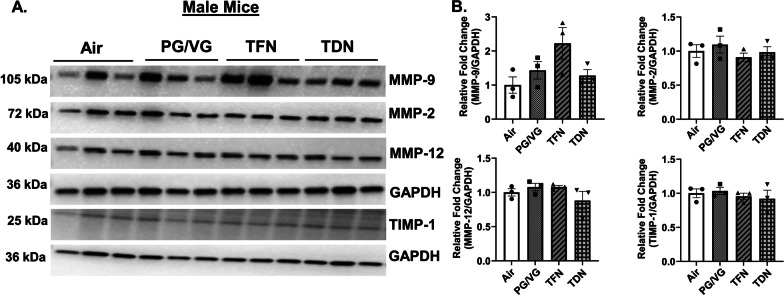
Differential effects of synthetic and tobacco-derived nicotine salts on MMPs protein abundance in male mice. Mice were exposed to air, PG/VG, PG/VG with TFN salts (TFN), and PG/VG with TDN salts (TDN) for five days for 1 h per day. Mice were sacrificed 2 h after final exposure. The protein abundance of MMP-2, MMP-9, MMP-12, and TIMP-1 was measured in lung homogenate with GAPDH used as a loading control by western blot. **A** Representative images for MMP-2, MMP-9, MMP-12, TIMP-1, and GAPDH for exposed male mice. **B** Band intensity was measured by densitometry with relative fold change being measured compared to air exposed male mice. Data represented as mean ± SEM and analyzed using one-way ANOVA with Tukey’s multiple comparison with *N* = 3/group. Images of full blots are shown in Additional file 1: Figs. S2B–S7B

In order to determine the effect of PG/VG with TFN and PG/VG with TDN salts to alter lung protease levels, protein abundance of MMPs was measured. In PG/VG exposure, both male and female mice had no change in MMP-2, MMP-9, MMP-12, or TIMP-1 protein abundance compared to air exposed mice (Figs. 3, 4). In TFN salt exposed mice, both female and male mice had no change in MMP-2, MMP-9, MMP-12, or TIMP-1 protein abundance compared to air exposed mice (Figs. 3, 4). In TDN salt exposed mice, both male and female mice had no change in MMP-2, MMP-12, or TIMP-1 protein abundance compared to control mice. Although, female mice exposed to TDN salts showed a significant increase in MMP-9 protein abundance as compared to air exposed mice while no change in the MMP-9 protein abundance was observed in male mice exposed to TDN salts compared to air controls (Figs. 3, 4).

### PG/VG with tobacco-derived nicotine salts alters MMP-9 and MMP-2 activity levels

**Fig. 5:**
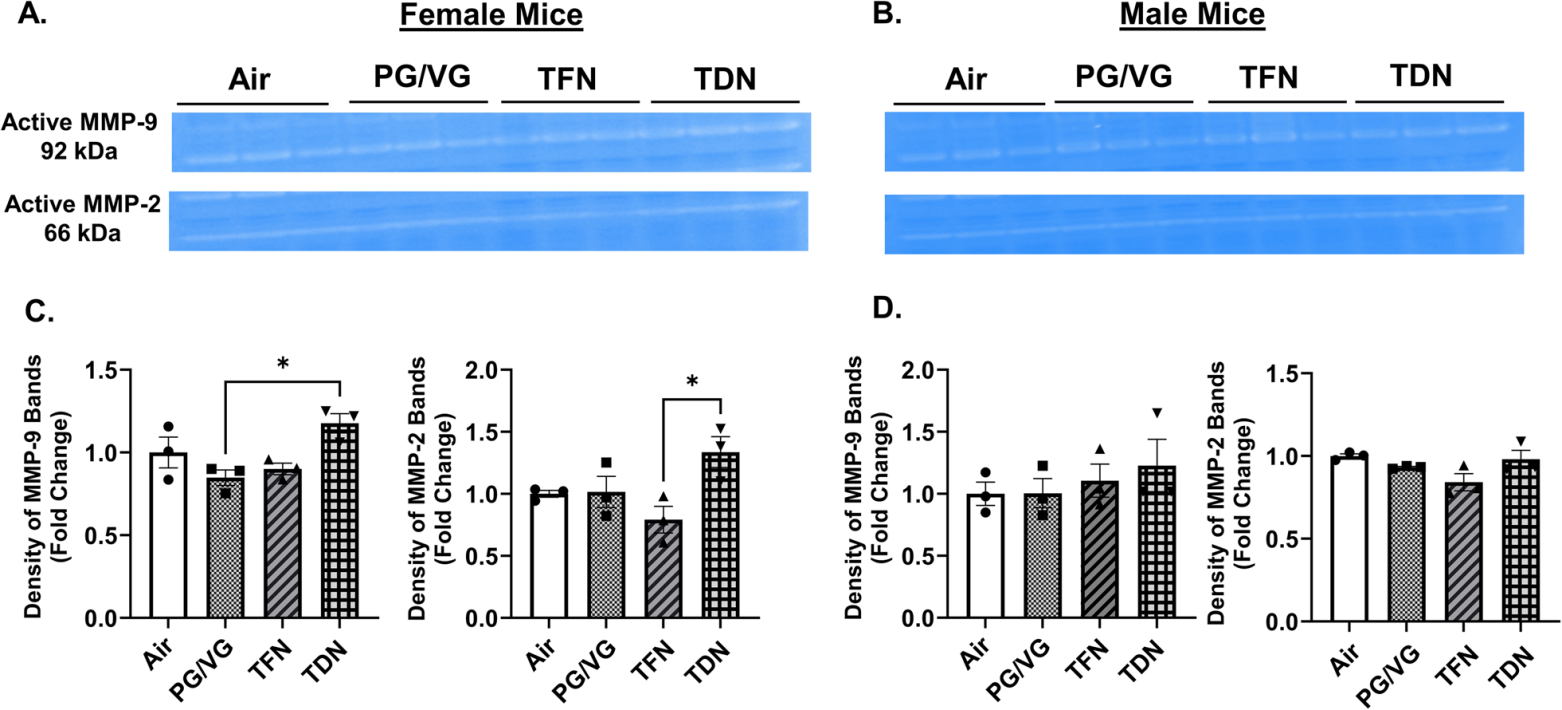
Differential effects of synthetic and tobacco-derived nicotine salts on MMP-9 and MMP-2 activity level. Mice were exposed to air, PG/VG, PG/VG with TFN salts (TFN), and PG/VG with TDN salts (TDN) for five days for 1 h per day. Mice were sacrificed 2 h after final exposure. The activity of MMP-2 and MMP-9 was measured in lung homogenate by gelatin gel zymography. Representative images for MMP-2 and MMP-9 for exposed (**A**) female and (**B**) male mice. Band intensity was measured by densitometry with relative fold change being measured compared to air exposed (**C**) female and (**D**) male mice. Data represented as mean ± SEM and analyzed using one-way ANOVA with Tukey’s multiple comparison with * *p* < 0.05, *N* = 3/group. Images of full gelatin gels are shown in Additional file 1: Fig. S8

In order to determine the effects of TFN and TDN salts to alter lung protease activity, total activity levels of MMP-2 and MMP-9 were determined using gel zymography. In PG/VG exposure, both male and female mice did not result in a significant change in MMP-2 or MMP-9 activity levels compared to air exposed mice (Fig. 5). In TFN salt exposed mice, both male and female mice did not result in a significant change in MMP-2 or MMP-9 activity levels compared to air exposed mice (Fig. 5). In TDN salt exposed mice, MMP-9 activity levels were significantly increased in female mice as compared to PG/VG only exposed mice group, whereas no change in the MMP-9 activity was observed in the males (Fig. 5). Furthermore, TDN salts exposure resulted in a significant increase in MMP-2 activity levels compared to TFN salts exposed female mice; while no change was observed for male mice (Fig. 5).

## Discussion

Although TFN has only recently begun to be used in tobacco products, TFN has been around for many years. However, unlike TDN, the health effects of TFN salts are relatively unknown [5, 16, 17, 22]. This study attempted to understand the potential toxicological differences between TDN and TFN salts. TFN salts and TDN salts were found to be at concentrations of roughly 50 mg/mL in e-liquids. Despite the similar exposures between the two exposure groups, there is a significantly increased level of serum cotinine in TDN salts exposed mice. This difference in serum cotinine levels indicates the possibility that TFN may be metabolized in mice differently than TDN. While there is limited knowledge about the metabolism and clearance of R- and S-cotinine in humans, a 1988 study showed the disposition kinetics of nicotine and cotinine enantiomers in rabbits and beagle dogs. This study showed that while the clearance of R- and S-nicotine differed in beagle dogs; the clearance of R-cotinine in rabbits was twice that of S-cotinine [17]. This substantiates our claim that biotransformation of the two enantiomers could be different for the TFN versus TDN and requires further research. Besides the differences in cotinine levels between TFN salts and TDN salts, serum cotinine levels in the air and PG/VG only exposure groups indicates that alteration in inflammatory cytokines and protease levels are due to exposure to the humectants alone.

Our results found that TFN salts increased total cell counts in the BALF but did not alter macrophage, neutrophil or eosinophil cell counts in BALF. While we looked into the myeloid cell population of immune cells in this study, there is a possibility that the exposures to TFN salts results in an increase in the lymphocytic (B-cell and T-cell) populations in these mice that could explain the significant increase in the total cell counts in mice exposed to TFN salts compared to controls. Future work in this area might be able to shed more light into this mechanism. Though not significant, the exposure to TFN salts resulted in a 1.6-fold increase in the neutrophil cell count in the lung homogenate as compared to the TDN salt exposed mouse lungs. This proves that the immunological responses on exposure to both TFN and TDN salts are distinct. Although our study found no change in eosinophil levels in either nicotine exposures, we observed a decrease in eosinophil count in lung digest of PG/VG exposed mice. This is contrary to previous observations. Chapman, et al., found that exposure of Balb/c mice to flavored e-liquids with nicotine resulted in a significant decrease in the eosinophil cell counts after treatment with house dust mites [23]. While Ahmad, et al., found that exposure of nicotine aerosol to male Sprague-Dawley rats led to elevated levels of eosinophil counts in the blood of exposed rats [24]. Although these exposures found contrary results, this may be due to different puffing topography, a higher temperature set for the heating of e-liquids, or the use of a nicotine solution not within PG or VG, indicating that the alterations in exposure methodologies can result in differential responses in rodents. Furthermore, it is also important to note that each of these studies used different rodent models and mouse strains which may not show comparable results as each strain has a distinct innate immune response [25].

Our results found that exposure to TDN salts and TFN salts had a significant decrease in IL-6, KC, and MCP-1 levels compared to exposure PG/VG alone exposed mice. Similar to these results, other investigations that utilized male Balb/c mice treated with lipopolysaccharide (LPS) by intratracheal administration and treated with nicotine by intraperitoneal administration, resulted in a decrease in IL-1, IL-6, and TNF-α cytokine levels in BALF compared to LPS treated alone mice [26]. Similarly, Glynos, et al. showed that exposure of male C57BL/6J mice to PG/VG with nicotine resulted in no alteration in cytokine levels of IL-1β, TNF-α, and IL-6 in the lung homogenate at an acute and sub-chronic exposure time points [27]. Although our study and previous work have shown anti-inflammatory effects of nicotine, contradictory evidences also exist. Ahmad, et al., found that male Sprague-Dawley rats exposed to nicotine aerosols results in an increase in the mRNA expression of IL-1α and CXCL1 along with increases in IL-1α protein levels [24]. Garcia-Arcos, et al. demonstrated an increase in the mRNA expression of IL-1β, MCP-1, and IL-6 in A/J mice exposed to PG/VG with nicotine as compared to PG/VG only [28]. These variations from our results and between previous studies could be a result of variations in the nicotine concentration, puffing topography and exposure duration. Further, these studies utilize freebase TDN while our study used both TDN and TFN salts for exposure which could explain the obtained results. Nevertheless, our results indicate that nicotine salts may have altered effects compared to freebase nicotine exposures.

PG/VG alone exposure resulted in an increase in cytokine levels of KC, IL-6, and MCP-1 in BALF. Previous studies have also shown a significant increase in IL-6 and IL-8 production by lung epithelial cells (16-HBE) exposed separately to propylene glycol or vegetable glycerin only [29], thus indicating that base humectants alone may pose a risk to e-cigarette users.

Our results showed that exposure to PG/VG and TFN salts did not alter the protein abundance of MMPs or TIMP-1 levels while TDN salts did increase MMP-9 protein abundance in female mice. A previous study has reported an increase in the mRNA expression of MMP-9 and MMP-12, along with other lung proteases in Cathepsin K and Cathepsin L1 in PG/VG with nicotine exposed study A/J mice as compare to only PG/VG exposures [28]. Similarly another study, demonstrated an increase in MMP-9 and MMP-12 levels in BALF of C57BL/6J mice exposed to PG/VG with nicotine [30].Similarly, Wang, et al. determined sex-specific alterations in the expressions of MMPs and TIMP-1 in pups born from pregnant dams exposed to PG/VG with nicotine, and found decrease in the levels of MMP-9 in both male and female offsprings [14]. Though the changes observed in this work were contrary to our results, but it still substantiates the fact that responses towards exposure to TFN and TDN salts varies based on sex, perinatal effects/pregnancy, and age (young/old) which should be an important consideration in future studies.

Previous work has also shown cell-specific changes in MMP production on e-cig aerosol exposure. In vitro exposure to flavored JUUL pods with 5% nicotine salts in lung epithelial cells (Beas2b) resulted in an upregulation of MMP-12 but downregulation of MMP-9 gene expression while in murine macrophages (Raw264.7) resulted in an upregulation in MMP-12 and no change in MMP-9 gene expression [31]. These alterations suggest the potential for lung extracellular matrix remodeling and the development of respiratory diseases caused by alterations in MMPs [32, 33]. In general, the expression and activity of MMPs varies in a spatial and inducible manner. The exact regulation of MMP production is not understood completely. However, they play a critical role in various biological processes including extracellular matrix remodeling and wound repair [34, 35]. Our results show a 17% and 33% increase in the MMP-9 and MMP-2 activity in the female mice exposed to TDN salts as compared to air controls. Of note, we also found a significant increase in the MMP-9 expression in the same group of mice in our study, thus showing sex-specific variation in MMP-9 activity in mouse exposed to TDN salts, but not TFN salts. Previous studies have associated MMP-9 with neutrophil migration on e-cig aerosol exposure [36]. Interestingly, we observed a 16% increase in the neutrophil counts in the BALF from TDN exposed mouse lungs in our study. However, due to the small sample size we did not study the sex-specific changes pertaining to the cell counts for this work. Future work is thus warranted with a larger sample size to understand the sex-specific variations in the MMP production and function on exposure to natural versus synthetic nicotine.

It is pertinent to mention here that there were few limitations in determining the source of nicotine used for TDN salts exposure in this study. Current analysis could definitively determine the presence of R- and S-nicotine in the e-liquids prepared using TFN salts but could only determine the presence of S-nicotine in the e-liquids prepared using TDN salts. Without conducting radiocarbon analysis to determine the C^14^ content of the nicotine, the e-liquids prepared using TDN salts cannot be determined to be tobacco-derived [5]. Furthermore, the duration of exposure for this study was too short to observe noticeable phenotypic changes within the lungs. Future work with longer exposure durations might be able to shed more light on the toxicity and immune-modulation on TFN exposures in vivo. Also, the sample size was small for this study to provide conclusive evidences for the sex-specific variations observed through our results. Future studies using more mouse numbers and with different mouse strains e.g. C57BL/6J vs. Balb/c along with human biological fluids from the users of TDN and TFN is imperative to understand the sex-, strain- and species-specific susceptibilities of TDN vs. TFN use.

Despite these shortcomings, this study is one of the first studies to determine the effects of exposure to TFN salts in mice and also compares the differences in the inhalation of S-nicotine and TFN. Besides being one of the first in vivo studies on TFN, this study utilized a nose-only exposure system and a puffing profile that mimics current e-cigarette user puffing topography, allowing this study to best mimic an exposure relevant to human users.

## Conclusions

Overall, this study was able to show that exposure to the humectants, PG/VG, used in e-cigarettes alone was able to increase cytokine levels, IL-6, KC, and MCP-1 in BALF and KC levels in lung homogenate of exposed C57Bl/6J mice. It also demonstrated that despite few changes in the differential immune cell counts in the BALF and lung homogenates, the level of KC, IL-6 and MCP-1 remained comparable in both TFN and TDN-exposed groups. We further provide evidence of sex-specific changes in the expression and activity of MMP-9 in TFN salt exposed mouse lungs. This study also suggests that the metabolism of nicotine or the clearance of cotinine from TFN may differ from the metabolism of nicotine or the clearance of cotinine from TDN. These findings indicate that exposure of humectants alone can induce an inflammatory response while exposure to TFN or TDN salts may differentially alter inflammatory responses and lung proteases production.

### Supplementary Information

**Additional file 1: Figure S1**. Differential effects of synthetic and tobacco-derived nicotine salts on infiltrating inflammatory cells in lung homogenates. **Figure S2**: Full images of MMP-9 for PG/VG with TFN salts and PG/VG with TDN salts exposure. **Figure S3**: Full images of MMP-2 for PG/VG with TFN salts and PG/VG with TDN salts exposure. **Figure S4**: Full images of MMP-12 for PG/VG with TFN salts and PG/VG with TDN salts exposure. **Figure S5**: Full images of GAPDH for MMP-9, MMP-2, and MMP-12 for PG/VG with TFN salts and PG/VG with TDN salts exposure. **Figure S6**: Full images of TIMP-1 for PG/VG with TFN salts and PG/VG with TDN salts exposure. **Figure S7**: Full images of GAPDH for TIMP-1 for PG/VG with TFN salts and PG/VG with TDN salts exposure. **Figure S8**: Full images of gelatin gels for PG/VG with TDN salts and PG/VG with TFN salts exposure.

## Data Availability

No datasets were generated or analysed during the current study.

## Abbreviations

ENDS: Electronic nicotine delivery systems
e-cigarettes: Electronic cigarette
PG: Propylene glycol
VG: Vegetable glycerin
TDN: Tobacco-derived nicotine
TFN: Tobacco-free or synthetic nicotine
BALF: Bronchoalveolar lavage fluid
MMP: Matrix metalloproteinases
IL: Interleukin
KC: Keratinocyte chemoattractant
MCP-1: Monocyte chemoattractant protein-1
FDA: Food and Drug Administration
^1^H NMR: Proton nuclear magnetic resonance
CO: Carbon monoxide
TPM: Total particulate matter
ELISA: Enzyme-linked immunosorbent assay
AO/PI: Acridine orange/propidium iodide
FBS: Fetal bovine serum
BSA: Bovine serum albumin
TBST: Tris-buffered saline with 0.1% tween 20
TIMP-1: Tissue inhibitor of metalloproteinases-1
GAPDH: Glyceraldehyde-3-phosphate dehydrogenase
mRNA: Messenger ribonucleic acid
AO/PI: Acridine orange/propidium iodide
LPS: Lipopolysaccharide
