# Supplementary material for: Tobacco-derived and tobacco-free nicotine cause differential inflammatory cell influx and MMP-9 in mouse lung
Source: Respir Res. 2024 Jan 23;25:51. doi: 10.1186/s12931-023-02662-5 (PMC10804532; doi:10.1186/s12931-023-02662-5)
Supplement: Supplementary file 1 — Additional file 1: Figure S1. Differential effects of synthetic and tobacco-derived nicotine salts on infiltrating inflammatory cells in lung homogenates. Figure S2: Full images of MMP-9 for PG/VG with TFN salts and PG/VG with TDN salts exposure. Figure S3: Full images of MMP-2 for PG/VG with TFN salts and PG/VG with TDN salts exposure. Figure S4: Full images of MMP-12 for PG/VG with TFN salts and PG/VG with TDN salts exposure. Figure S5: Full images of GAPDH for MMP-9, MMP-2, and MMP-12 for PG/VG with TFN salts and PG/VG with TDN salts exposure. Figure S6: Full images of TIMP-1 for PG/VG with TFN salts and PG/VG with TDN salts exposure. Figure S7: Full images of GAPDH for TIMP-1 for PG/VG with TFN salts and PG/VG with TDN salts exposure. Figure S8: Full images of gelatin gels for PG/VG with TDN salts and PG/VG with TFN salts exposure. [file 12931_2023_2662_MOESM1_ESM.pdf]

Additional file 1

Tobacco-Derived and Tobacco-Free Nicotine cause differential inflammatory cell influx and MMP-9 levels in mouse lung

Thomas Lamb, Gagandeep Kaur, and Irfan Rahman

[1]Department of Environmental Medicine, University of Rochester Medical Center, Rochester, NY, USA

Figure S1

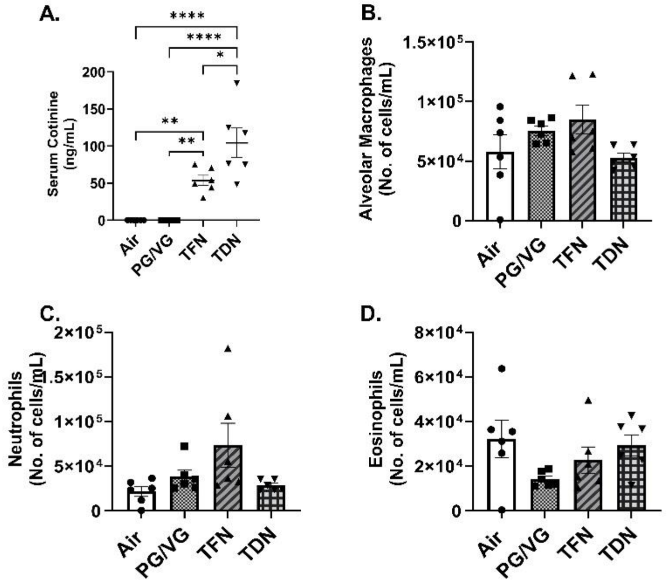

Figure S1: Differential effects of synthetic and tobacco-derived nicotine salts on infiltrating inflammatory cells in lung homogenates. Mice were exposed to air, PG/VG, PG/VG with TFN salts (TFN), and PG/VG with TDN salts (TDN) for five days for one hour per day. Mice were sacrificed two hours after final exposure. (A) Serum cotinine was determine using ELISA-based cotinine assay. Flow cytometry was performed to determine the number of (B) Alveolar Macrophages ($CD45^{+}$Siglec $F^{+}CD11b^{-}$), (C) Neutrophils ($CD45^{+}$Siglec $F^{-}CD11b^{+}Ly6G^{+}$), (D) Eosinophils ($CD45^{+}CD11b^{+}Ly6G^{-}CD11c^{-}$ Siglec F^{+}) in lung homogenate of control and experimental groups. Data represented as mean ± SEM and analyzed using one-way ANOVA with Tukey’s multiple comparison with * $p < 0.05$, ** $p < 0.01$, and **** $p < 0.0001$, N = 6.

Figure S2

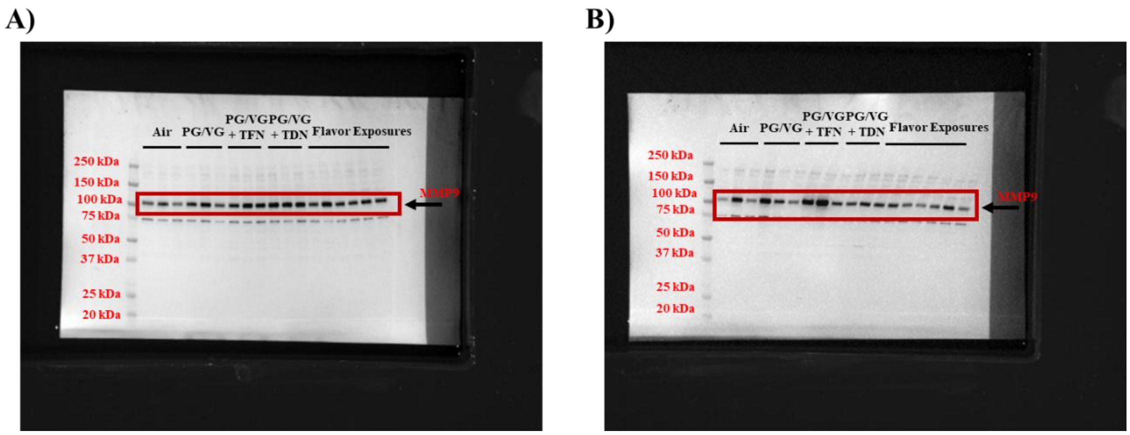

Figure S2: Full images of MMP-9 for PG/VG with TFN salts and PG/VG with TDN salts exposure. Images represent the full blot for MMP9 with (A) female mice and (B) male mice blots representing the image after each membrane was probed for MMP-2, MMP-12 and then stripped and then probed with MMP-9 (dilution 1:1000). Flavor Exposure as denoted in this blot are other sample group from the same study which is not discussed in this manuscript or pertaining to this manuscript.

Figure S3

A) B)

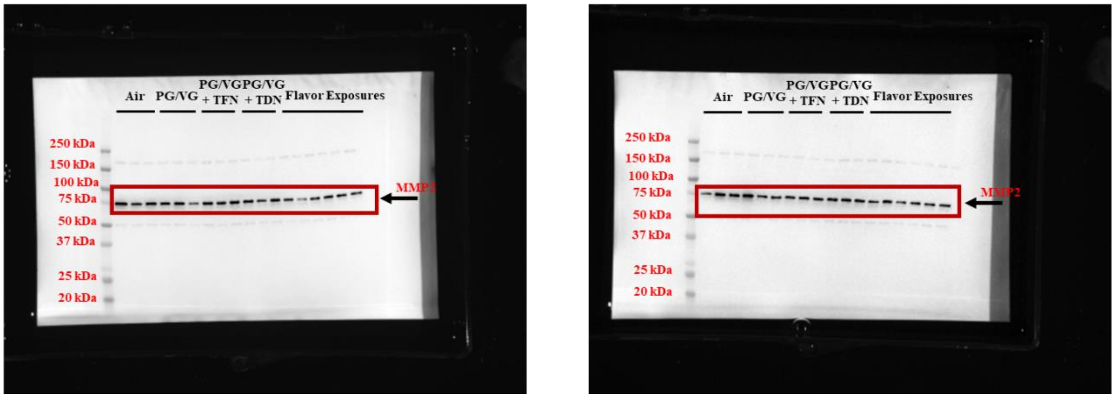

Figure S3: Full images of MMP-2 for PG/VG with TFN salts and PG/VG with TDN salts exposure. Images represent the full blot for MMP-2 with (A) female mice and (B) male mice blots representing the image after each membrane was probed for MMP-2 (dilution 1:1000). Flavor Exposure as denoted in this blot are other sample group from the same study which is not discussed in this manuscript or pertaining to this manuscript.

Figure S4

A) B)

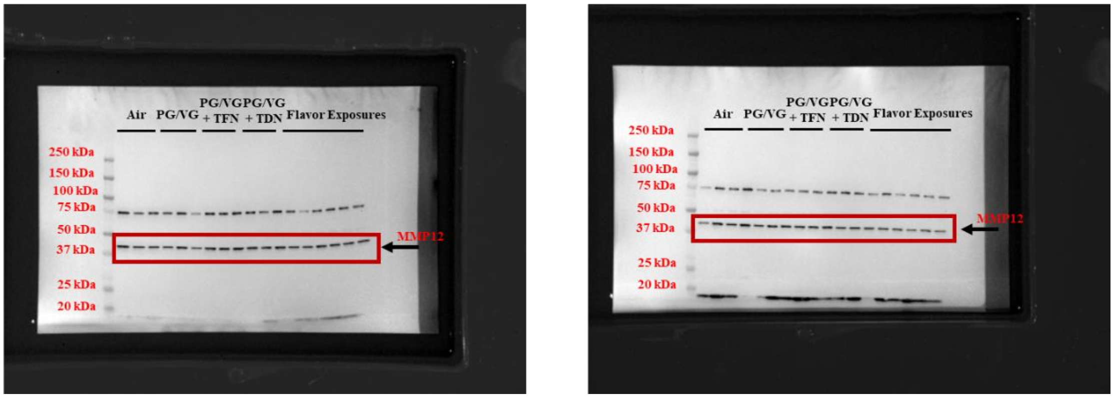

Figure S4: Full images of MMP-12 for PG/VG with TFN salts and PG/VG with TDN salts exposure. Images represent the full blot for MMP-12 with (A) female mice and (B) male mice blots representing the image after each membrane was probed for MMP-2 and then stripped and then probed with MMP-12 (dilution 1:1000). Flavor Exposure as denoted in this blot are other sample group from the same study which is not discussed in this manuscript or pertaining to this manuscript.

Figure S5

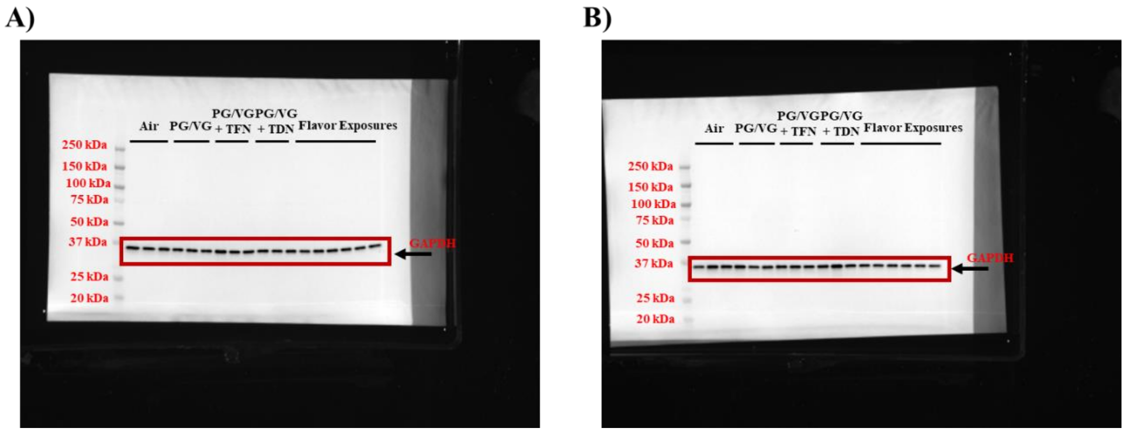

Figure S5: Full images of GAPDH for MMP-9, MMP-2, and MMP-12 for PG/VG with TFN salts and PG/VG with TDN salts exposure. Images represent the full blot for GAPDH with (A) female mice and (B) male mice blots representing the image after each membrane has been probed for MMP-2, MMP-12, MMP-9 and then stripped and finally probed with GAPDH (dilution 1:1000). Flavor Exposure as denoted in this blot are other sample group from the same study which is not discussed in this manuscript or pertaining to this manuscript.

Figure S6

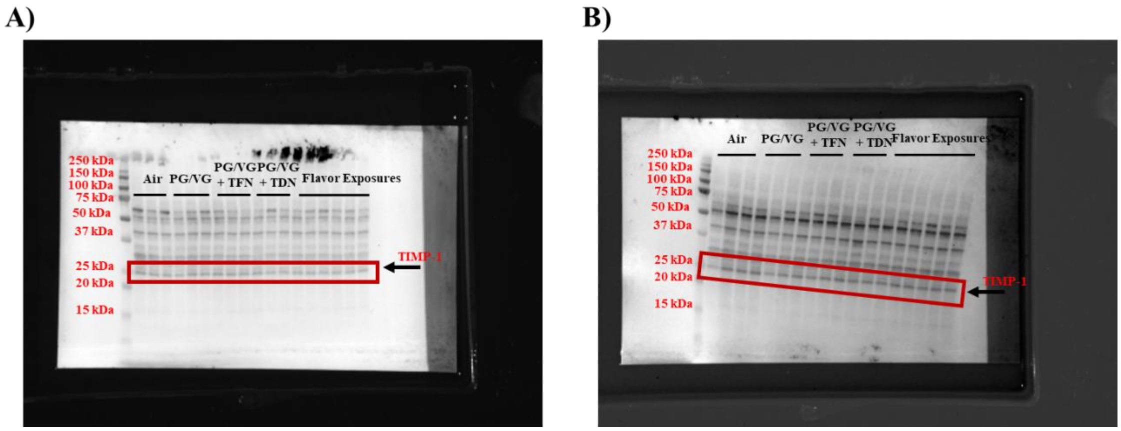

Figure S6: Full images of TIMP-1 for PG/VG with TFN salts and PG/VG with TDN salts exposure. Images represent the full blot for TIMP-1 with (A) female mice and (B) male mice blots representing the image after each membrane was probed for TIMP-1 (dilution 1:1000). Flavor Exposure as denoted in this blot are other sample group from the same study which is not discussed in this manuscript or pertaining to this manuscript.

Figure S7

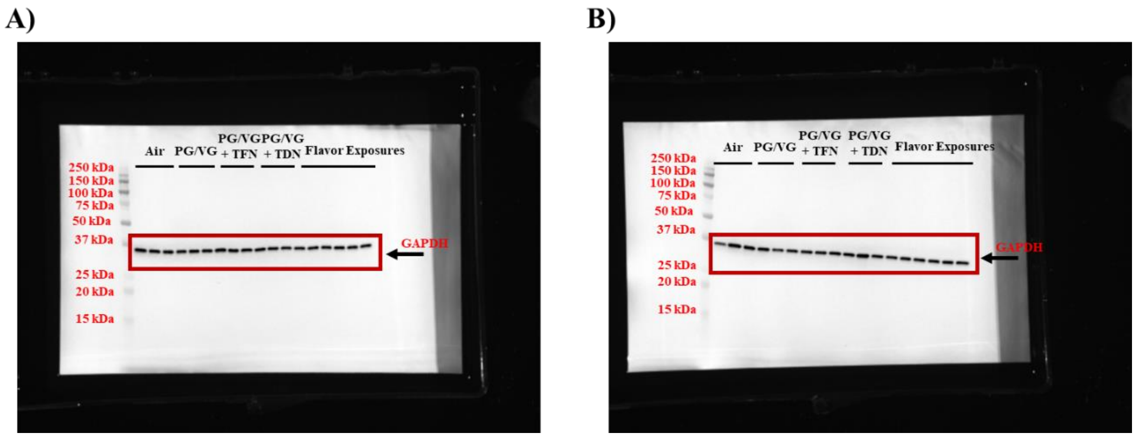

Figure S7: Full images of GAPDH for TIMP-1 for PG/VG with TFN salts and PG/VG with TDN salts exposure. Images represent the full blot for GAPDH with (A) female mice and (B) male mice blots representing the image after each membrane was probed for TIMP-1 and then stripped and finally probed with GAPDH (dilution 1:1000). Flavor Exposure as denoted in this blot are other sample group from the same study which is not discussed in this manuscript or pertaining to this manuscript.

Figure S8

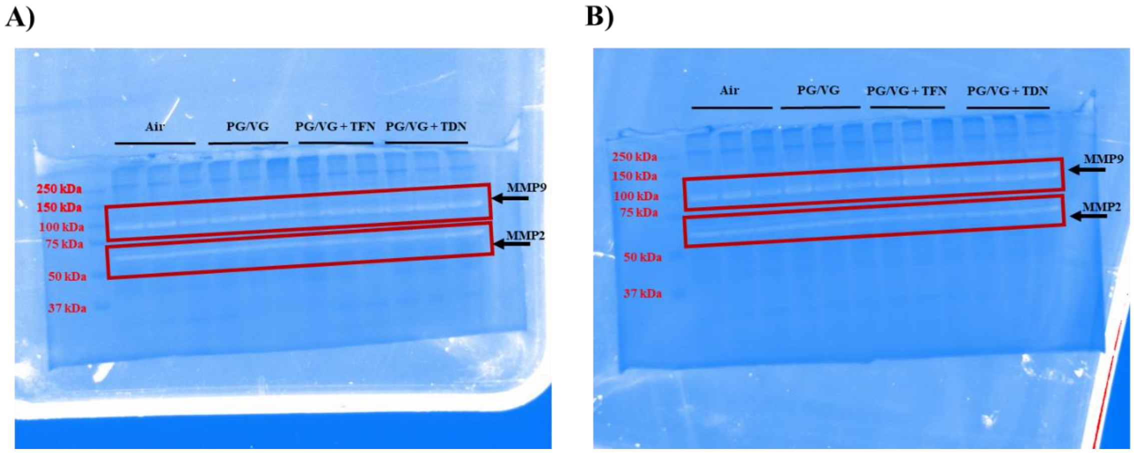

Figure S8: Full images of gelatin gels for PG/VG with TDN salts and PG/VG with TFN salts exposure. Images represent the full gelatin gel for MMP-9 and MMP-2 activity levels with the images for the (A) female mice and (B) male mice gel after being stained for one hour and then incubated with destaining solution.
